# Search for biocontrol agents among endophytic lipopeptide-synthesizing bacteria Bacillus spp. to protect wheat plants against Greenbug aphid (Schizaphis graminum)

**DOI:** 10.18699/vjgb-24-32

**Published:** 2024-06

**Authors:** S.D. Rumyantsev, V.Y. Alekseev, A.V. Sorokan, G.F. Burkhanova, E.A. Cherepanova, I.V. Maksimov, S.V. Veselova

**Affiliations:** Institute of Biochemistry and Genetics of the Ufa Federal Research Centre of the Russian Academy of Sciences, Ufa, Russia; Institute of Biochemistry and Genetics of the Ufa Federal Research Centre of the Russian Academy of Sciences, Ufa, Russia; Institute of Biochemistry and Genetics of the Ufa Federal Research Centre of the Russian Academy of Sciences, Ufa, Russia; Institute of Biochemistry and Genetics of the Ufa Federal Research Centre of the Russian Academy of Sciences, Ufa, Russia; Institute of Biochemistry and Genetics of the Ufa Federal Research Centre of the Russian Academy of Sciences, Ufa, Russia; Institute of Biochemistry and Genetics of the Ufa Federal Research Centre of the Russian Academy of Sciences, Ufa, Russia; Institute of Biochemistry and Genetics of the Ufa Federal Research Centre of the Russian Academy of Sciences, Ufa, Russia

**Keywords:** Bacillus spp., Schizaphis graminum, endophytes, PCR, RT-PCR, plant-microbial interactions, lipopeptides, biological control agents, Bacillus spp., Schizaphis graminum, эндофитные бактерии, ПЦР, ПЦР в реальном времени, растительно-микробные взаимодействия, липопептиды, биопрепараты

## Abstract

Beneficial endophytic bacteria can suppress the development of insect pests through direct antagonism, with the help of metabolites, or indirectly by the induction of systemic resistance through the regulation of hormonal signaling pathways. Lipopeptides are bacterial metabolites that exhibit direct antagonistic activity against many organisms, including insects. Also, lipopeptides are able to trigger induced systemic resistance (ISR) in plants against harmful organisms, but the physiological mechanisms of their action are just beginning to be studied. In this work, we studied ten strains of bacteria isolated from the tissues of wheat and potatoes. Sequencing of the 16S rRNA gene showed that all isolates belong to the genus Bacillus and to two species, B. subtilis and B. velezensis. The genes for lipopeptide synthetase – surfactin synthetase (Bs_srf ), iturin synthetase (Bs_ituA, Bs_ituB) and fengycin synthetase (Bs_fenD) – were identified in all bacterial isolates using PCR. All strains had high aphicidal activity against the Greenbug aphid (Schizaphis graminum Rond.) due to the synthesis of lipopeptides, which was proven using lipopeptide-rich fractions (LRFs) isolated from the strains. Endophytic lipopeptide-synthesizing strains of Bacillus spp. indirectly affected the viability of aphids, the endurance of plants against aphids and triggered ISR in plants, which manifested itself in the regulation of oxidative metabolism and the accumulation of transcripts of the Pr1, Pr2, Pr3, Pr6 and Pr9 genes due to the synthesis of lipopeptides, which was proven using LRF isolated from three strains: B. subtilis 26D, B. subtilis 11VM, and B. thuringiensis B-6066. We have for the first time demonstrated the aphicidal effect of fengycin and the ability of the fengycin-synthesizing strains and isolates, B. subtilis Ttl2, Bacillus sp. Stl7 and B. thuringiensis B-6066, to regulate components of the pro-/antioxidant system of aphid-infested plants. In addition, this work is the first to demonstrate an elicitor role of fengycin in triggering a systemic resistance to S. graminum in wheat plants. We have discovered new promising strains and isolates of endophytes of the genus Bacillus, which may be included in the composition of new biocontrol agents against aphids. One of the criteria for searching for new bacteria active against phloem-feeding insects can be the presence of lipopeptide synthetase genes in the bacterial genome.

## Abstract (ru)

Полезные эндофитные бактерии могут подавлять развитие вредителей за счет прямого антаго- низма, с помощью метаболитов или опосредованно индуцировать системную устойчивость через регуляцию гормональных сигнальных путей. Липопептиды – бактериальные метаболиты, проявляющие прямую антаго- нистическую активность ко многим организмам, в том числе к насекомым. Также липопептиды способны за- пускать системную индуцированную устойчивость у растений против вредных организмов. В настоящее время механизм действия бактериальных метаболитов липопептидов на защитную систему растений только начи- нают исследовать. В данной работе изучено десять штаммов и изолятов бактерий, выделенных из внутренних тканей культурной и дикой пшеницы и картофеля. Секвенирование гена 16S рРНК показало принадлежность всех изолятов к роду Bacillus и двум видам – B. subtilis и B. velezensis. У всех бактериальных изолятов методом ПЦР были идентифицированы гены липопептид синтаз – сурфактин синтазы (Bs_srf ), итурин синтаз (Bs_ituA, Bs_ituB) и фенгицин синтазы (Bs_fenD). Все штаммы обладали афицидной активностью в отношении обыкновен- ной злаковой тли (Schizaphis graminum Rond.) за счет синтеза липопептидов, что было доказано с помощью ли- попептид-богатых фракций (ЛБФ), выделенных из штаммов. Эндофитные липопептид-синтезирующие штаммы Bacillus spp. опосредованно влияли на жизнеспособность тли, выносливость растений по отношению к тле и запускали системную индуцированную устойчивость у растений, что проявлялось в регуляции окислительно- го метаболизма и накоплении транскриптов генов Pr1, Pr2, Pr3, Pr6 и Pr9, за счет синтеза липопептидов, что подтверждено с помощью ЛБФ, выделенных из трех штаммов – B. subtilis 26D, B. subtilis 11VM и B. thuringiensis B-6066. В нашей работе впервые показано афицидное действие фенгицина и способность штаммов и изолятов B. subtilis Ttl2, Bacillus sp. Stl7 и B. thuringiensis B-6066, синтезирующих фенгицин, регулировать компоненты про-/антиоксидантной системы растений, зараженных тлей. Кроме того, впервые продемонстрирована элиситорная роль фенгицина в запуске системной устойчивости растений пшеницы к S. graminum. Обнаружены новые перспективные штаммы и изоляты эндофитных бактерий рода Bacillus, которые могут стать основой будущих биопрепаратов против тлей. Одним из критериев поиска новых бактерий, активных против насекомых, питающихся флоэмным соком, может быть наличие в бактериальном геноме генов липопептид синтаз.

## Introduction

Insects of the order Hemiptera, aphids, whiteflies, planthoppers, including the Greenbug aphid Schizaphis graminum, which are sap-sucking insects, can cause severe yield losses of up to 60–80 % due to their influence on photosynthesis processes and biomass growth rate (Koch et al., 2016; Radchenko et al., 2022). Currently, chemical insecticides remain the main agents of controlling phloem-feeding pests, leading to the emergence of new pesticide-resistant forms of pests. Therefore, it is necessary to find environmentally friendly biological control agents to defend plants from pests. Such effective biological control agents can be endophytic growthpromoting bacteria that can live inside plants without causing diseases in them (Rani et al., 2022).

Currently, many researchers suppose that endophytes protect plants from stress through the mechanisms of direct or indirect protective effects on harmful organisms due to the synthesis and secretion of diverse metabolites (Oukala et al., 2021; Xia et al., 2022). The direct action of endophytes is carried out due to the biocidal activity of some metabolites (bacteriocins, biosurfactants, lipopeptides). Indirect action is expressed in the ability of endophytes to stimulate growth processes in plants, improve the immune system of plants, and build a durable defense of the host against harmful organisms, which is known as priming (Rashid, Chung, 2017; Xia et al., 2022). Bacteria-induced priming provides faster and longer-lasting plant protection throughout the growing season with low physiological costs, making endophyte-based biocontrol agents very promising (Oukala et al., 2021; Rani et al., 2022; Xia et al., 2022). Activation of the plant immune system and priming by endophytes is realized by triggering induced systemic resistance (ISR) against harmful organisms, which has been shown by many researchers and summarized in recent reviews (Oukala et al., 2021; Rani et al., 2022; Xia et al., 2022). Endophyte-activated ISR is regulated by bacterialproduced hormone-like substances with growth-regulating activity such as abscisic (ABA), salicylic (SA), jasmonic acids (JA), and ethylene (ET) (Pieterse et al., 2014; Rashid, Chung, 2017). The characteristic features of ISR are jumps in the generation of reactive oxygen species (ROS) and changes in the gene expression with a focus on defense-related genes of pathogenesis-related proteins (PR proteins) (Oukala et al., 2021; Xia et al., 2022).

Bacteria of Bacillus spp. are famous for their ability to synthesize a wide range of diverse metabolites (Miljaković et al., 2020). Bacterial metabolites are the active ingredient of any biocontrol agent. Lipopeptides are one of the major classes of bacterial metabolites intensively researched in recent years. Lipopeptides are small peptides that have biocidal properties against mycoplasmas, bacteria, yeasts, fungi, oomycetes, nematodes, and pests due to their capability to connect to the lipid bilayer of the plasmalemma and change its permeability (Andrić et al., 2021). Bacteria of the genus Bacillus produce lipopeptides of three families: surfactins, fengycins and iturins (Andrić et al., 2021). Recently, the insecticidal activity of lipopeptides against the orders Diptera, Coleoptera, Hemiptera, and Lepidoptera have been shown in some studies (Rodríguez et al., 2018; Denoirjean et al., 2022). Currently, the eliciting role of lipopeptides in triggering systemic resistance in plants is being actively studied (Rashid et al., 2018; Tunsagool et al., 2019; Miljaković et al., 2020). The elicitor role of lipopeptides against a wide range of pathogens of plants has been shown in many studies (Tunsagool et al., 2019; Jiang et al., 2021). However, information on the elicitor role of lipopeptides in triggering ISR in plants against sucking insects is limited (Rashid et al., 2018; Rumyantsev et al., 2023).

Thus, the search for highly effective endophytic strains for plant protection against sap-sucking insects using the priming mechanism, the study of the metabolic composition and mechanisms of action of endophytes is an urgent task. In this regard, the aim of our work was to study the elicitor role of lipopeptides and the ability of endophytic bacteria that synthesize lipopeptides to protect plants through the priming mechanism. To do this, in our work we searched for strains and isolates of the genus Bacillus capable of synthesizing lipopeptides, studied the insecticidal activity of bacteria in relation to Greenbug aphid, and also studied the indirect effect of endophytes and lipopeptide-rich fractions (LRFs) of three strains – B. subtilis 26D, B. subtilis 11VM and B. thuringiensis B-6066 – on the redox status, indicators of resistance (antibiosis and endurance) to the pest, and changes in the expression of defense-related genes of PR proteins of wheat plants populated by S. graminum.

## Materials and methods

Bacteria, plants and insects. In this work, gram-positive aerobic endophytic bacteria from the collection of the Laboratory of Biochemistry of Plant Immunity of the Institute of Biochemistry and Genetics of the Ufa Federal Research Centre of the Russian Academy of Sciences (UFRC RAS) were used. Three strains of Bacillus subtilis, 26D (Russian Collection of Agricultural Microorganisms (RCAM), No. 128), 11VM (RCAM No. 519), Ttl2 (isolated from the leaves of Triticum timopheevii Zhuk., Republic of Bashkortostan), one strain of B. thuringiensis, B-6066 (All-Russian collection of industrial microorganisms (ARCIM), No. 6066), and six isolates of Bacillus spp. isolated from leaves of wheat and potatoes growing on the territory of the Republic of Bashkortostan were used. Bacteria were grown on liquid lysogenic broth (LB) medium (1 % tryptone, 0.5 % yeast extract and 0.5 % NaCl) in 50 ml flasks at 28 °C using laboratory shakers (120 rpm) within 72 h until complete sporulation

In this work, we studied the population of Greenbug aphid (Schizaphis graminum Rond.), 2020, which was maintained under laboratory-controlled conditions on plants of common spring wheat (Triticum aestivum L.) cv. Salavat Yulaev (SY) as described previously (Rumyantsev et al., 2023). Seeds of cv. SY were obtained from the Bashkir Research Institute of Agriculture – Subdivision of the UFRC RAS.

Isolation of DNA from bacteria. Genomic DNA from bacteria was isolated with a lysis buffer containing 1 % Chelex 100 resin (BioRad Laboratories, USA), as described earlier (Veselova et al., 2022).

16S rRNA gene sequencing. The gene of 16S rRNA was amplified using the universal primers 27F (5′-CAGAGTTT GATCCTGGCT-3ʹ) and 1492R (5′-AGGAGGTGATCCAG CCGCA-3ʹ). Amplified fragments of the 16S RNA gene of Bacillus spp. isolates were visualized on a 1 % agarose gel stained with ethidium bromide. Then, PCR fragments of the 16S RNA gene were excised from the agarose gel and purified using a diaGene agarose gel DNA elution kit (DiaM, Russia). Sanger sequencing of PCR fragments was performed on a 3500xL genetic analyzer from Applied Biosystems (Evrogen, Russia). BLAST software was used for alignment and comparison of the obtained sequences of Bacillus spp. isolates with sequences deposited in GenBank. These results were used for identifying what matched the searched sequence and what species the isolates under consideration belonged to. Data on sequences and species of bacteria were submitted in GenBank (see Table 3).

**Table 3.**
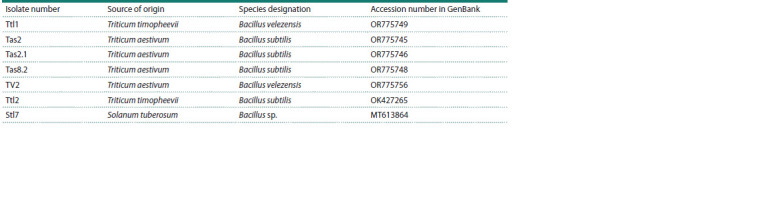
Characterization of bacteria isolated from the inner tissues of plants

Detection of genes of lipopeptide synthetase in the Bacillus spp. strains and isolates by PCR. The genes of lipopeptide synthetase – surfactin synthetase (srf ), iturin synthetases (ituA, ituB) and fengycin synthetase ( fenD) – were identified in bacterial strains and isolates using PCR with gene-specific primers. Primers to the bac gene encoding 16S RNA of Bacillus spp. were used as an inner control. The sequences of all primers are presented in Table 1.

**Table 1.**
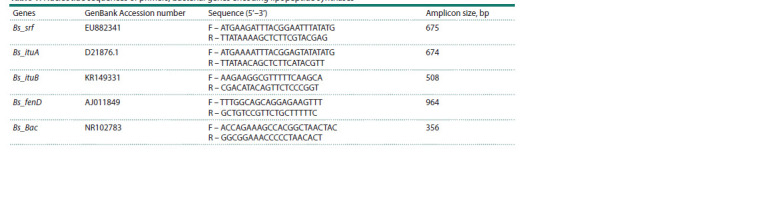
Nucleotide sequences of primers, bacterial genes encoding lipopeptide synthases

Isolation of the lipopeptide-rich fraction (LRF) from the Bacillus spp. strains. LRFs from the acidified liquid bacterial culture medium of three Bacillus spp. strains B. subtilis 11VM, B. subtilis 26D and B. thuringiensis B-6066 and two isolates Bacillus sp. Tas2.1 and Bacillus sp. Tas8.2 were obtained by ethanol extraction followed by purification on an Amicon Ultracel-3K filter (Merck KGaA, Darmstadt, Germany) as described previously (Maksimov et al., 2020). The purified lipopeptide fraction was weighed and dissolved in 80 % ethanol, the growth-promoting concentrations selected earlier were used (Maksimov et al., 2020).

Aphicidal activity of the Bacillus spp. strains and isolates. Aphicidal activity of bacterial strains and LRF was studied on first cut leaves of wheat seedlings cv. SY, placed in test tubes with 5 ml of the bacterial suspension at the concentration of 107 cells/ml (control tubes contained 5 ml of sterile water) or those with 5 ml of LRF at various concentrations from 2.5 to 200 μg/ml according to a method modified for wheat and described earlier (Veselova et al., 2019). Aphicidal activity was expressed as mortality rate (%) among the total number of aphids (Veselova et al., 2019).

Experimental conditions. Before planting, some wheat seeds were treated with a liquid culture of bacteria in a semidry manner at growth-stimulating concentrations selected earlier (Alekseev et al., 2021; Rumyantsev et al., 2023). The cell titer in the suspension was counted at 600 nm using a SmartSpectm Plus spectrophotometer (Bio-Rad, USA) certified for this task. The cell titer of the studied cultures was (1.8–2)·109 cells/ml; by adding distilled water, the suspensions were diluted to a final titer of (2–4)·106 cells/ml and the resulting suspensions were used for seed treatment. The final titer of B. subtilis 26D, Bacillus sp. Tas2.1 and B. thuringiensis B-6066 was 4·106 cells/ml. The final titer of Bacillus sp. Tas8.2, B. subtilis 11VM, B. subtilis Ttl2 and Bacillus sp. Stl7 was 2·106 cells/ml.

Wheat seedlings were grown in 1-liter vessels on an aquatic culture (10 % Hoagland–Arnon solution) under aphid breeding conditions. Solutions of LRFs at growth-stimulating concentrations selected earlier (Alekseev et al., 2021) were added to the plant nutrient medium 24 h before aphid colonization. After 24 h, the medium was replaced with Hoagland–Arnon solution without LRFs. Growth-promoting concentrations of LRF of Bacillus spp. strains B. subtilis 11VM, B. subtilis 26D and B. thuringiensis B-6066 and isolates Bacillus sp. Tas2.1 and Bacillus sp. Tas8.2 were 1.5, 2.5, 1.5, 2.5 and 2.0 μg/ml, respectively (Alekseev et al., 2021). Plant treatment with LRFs was carried out to establish the elicitor role of lipopeptides in the induction of defensive signaling pathways in plants and did not pursue the goal of studying these metabolites as independent biocontrol agents. The colonization of 4-day-old wheat seedlings by aphids was carried out as described earlier (Rumyantsev et al., 2023).

Antibiosis test and endurance test. The antibiosis test was carried out as described earlier (Veselova et al., 2019). Mortality and fecundity of aphids were expressed as % of the total number of aphids. The propagation coefficient was calculated as described earlier (Veselova et al., 2019). Plant endurance was assessed by measuring the length of the first and second leaves of seedlings as described previously and expressed as % leaf growth compared to uninfected control (Veselova et al., 2019).

The content of hydrogen peroxide (H2O2) and the activity of enzymes – peroxidase (POD) and catalase (CAT) were analyzed according to standard methods (Rumyantsev et al., 2023). To measure the content of hydrogen peroxide (H2O2) and enzyme activity, plant material was homogenized 24 and 72 hours after colonization by S. graminum in 0.05 M Na-phosphate buffer (PB), pH 6.2, in a ratio of 1:5 (wt/vol) and incubated at 4 °C for 30 min. The supernatant was separated by centrifugation at 15,000 g for 15 min (5415K Eppendorf, Germany). The concentration of Н2О2 in the supernatant was determined according to the method of (Bindschedler et al., 2001; Maksimov et al., 2011), using orange xylenol in the presence of Fe2+ ions. After coloring, the mixture was centrifuged for 5 min at 10,000 g and the optical density was measured at a wavelength of 560 nm on an LS 55 Luminescence Spectrometer (Perkin Elmer, USA). H2O2 content was calculated using a calibration curve and expressed in μmol H2O2/g fresh weight (FW). POD activity was determined by a micromethod in 96-well plates (Corning-Costar, USA) by the oxidation of (o-)phenylenediamine in the presence of H2O2 at 490 nm on a Benchmark Microplate Reader spectrophotometer (Bio-Rad Laboratories, USA) (Veselova et al., 2014). The enzyme activity was expressed in optical density/mg protein per minute, which corresponded to the amount of oxidized substrate causing an increase in optical density in 1 min. CAT activity was determined by a micromethod based on the ability of H2O2 to form a stable colored complex with molybdate salts (Veselova et al., 2014). Optical density was measured at 405 nm on a Benchmark Microplate Reader spectrophotometer. CAT activity was calculated using a calibration curve and expressed in μmol H2O2/(mg protein per min). Protein content was determined by the Bradford method.

Performing qPCR. Isolation of RNA from wheat leaves (five plants per repeat) fixed in liquid nitrogen 1, 3, and 6 days after aphid infestation was performed using Lira® (Biolabmix, Russia) according to the manufacturer’s instructions. cDNA synthesis was performed as described previously (Veselova et al., 2022). Expression of genes encoding PR proteins was analyzed by quantitative real-time PCR using a CFX Connect real-time PCR Detection System device (BioRad Laboratories, USA) and a set of predefined reagents EvaGreen I (Sintol, Russia). In the work, primers for the genes encoding PR1 protein, PR2 protein – glucanase, PR3 protein – chitinase, PR6 protein – proteinase inhibitors and PR9 protein peroxidase were used. To standardize the data, the wheat gene TaRli (RNaseLinhibitor-like) was used as an inner reference for the real-time qPCR analysis. Primers for qRT-PCR were designed using a web-based primer designing tool from IDT (http://eu.idtdna.com/Scitools/Applications/Primerquest) (USA). Primer sequences were validated by the presence of only a single peak on the thermal dissociation (Tm) curve generated by the thermal denaturing protocol. The sequences of all primers are presented in Table 2.

**Table 2.**
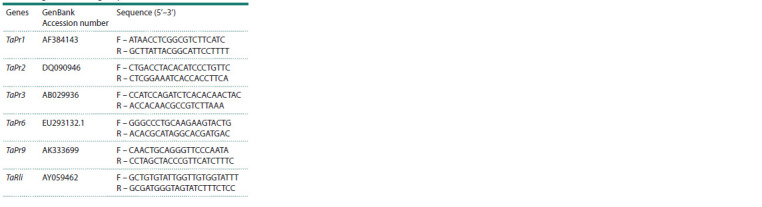
Nucleotide sequences of primers for wheat genes encoding PR-proteins

To quantify relative gene expression, the delta-delta Ct method was applied using the CFX Connect real-time PCR Detection System (BioRad Laboratories, USA) as described previously (Veselova et al., 2022). Three independent biological and three technical replications were performed for each experiment

Statistical analysis. The experiments were carried out in triplicate with a different number of biological repetitions, from 3 to 10, depending on the type of analysis. The exact number of replicates for each analysis is indicated in the table note or figure legend. Experimental data were expressed as means ± SE, which were calculated in all treatments using MS Excel. The significance of differences was assessed by ANOVA followed by Duncan’s test ( p ≤ 0.05) with STATISTICA 10.0 software

## Results

Characterization of bacteria isolated from the inner tissues of plants

Two strains, B. subtilis 26D and B. subtilis 11VM, were used in the work as reference endophytic strains with known properties and protective action against Greenbug aphid (Rumyantsev et al., 2023). Previously, it was shown that the B. subti-lis 26D strain synthesizes surfactin, and the B. subtilis 11VM strain synthesizes iturin (Rumyantsev et al., 2023). B. thuringiensis B-6066 also induced resistance against aphids, but was not tested for the ability to synthesize lipopeptides (Veselova et al., 2019).

Two isolates from the UFRC RAS collection of microorganisms were previously sequenced using 16S RNA gene fragments: Bacillus sp. Stl7 (GenBank: MT613864) (isolated from the inner tissues of leaves of Solanum tuberosum L., Republic of Bashkortostan) and B. subtilis Ttl2 (GenBank: OK427265) (Sorokan et al., 2020; Veselova et al., 2022) (Table 3). For the remaining five isolates, presented in Table 3, fragments of the 16S RNA gene were sequenced in this work. Isolate of Bacillus sp. Ttl1 was isolated from the inner tissues of the leaves of T. timopheevii, the remaining isolates of Bacillus sp. Tas2, Tas8.2, TV2 and Tas2.1 were isolated from the inner tissues of common spring wheat leaves (T. aestivum) (Table 3). Isolates of Bacillus sp. Ttl1 and TV2 were designated as Bacillus velezensis. Isolates of Bacillus sp. Tas2, Tas2.1, Tas8.2 were designated as B. subtilis (Table 3).

Detection of genes of lipopeptide synthetases in the Bacillus spp. strains and isolates

**Fig. 1.**
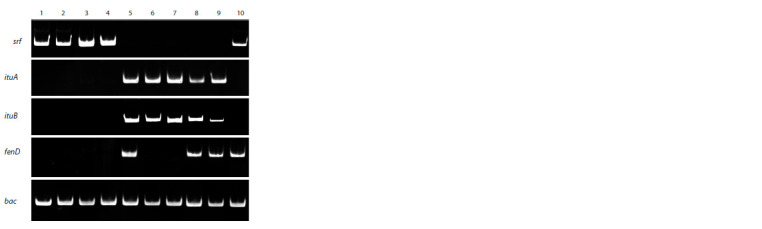
PCR analysis of bacteria Bacillus spp. for the presence of lipopeptide synthetase genes: srf – surfactin synthetase, ituA and ituB – iturin synthetase, and fenD – fengycin synthetase, bac – reference gene. The samples are indicated as follows: 1 – B. subtilis 26D; 2 – B. subtilis Tas2; 3 – B. subtilis Tas8.2; 4 – B. subtilis Tas2.1; 5 – B. subtilis11VM; 6 – B. velezensis TV2; 7 – B. velezensis Ttl1; 8 – B. subtilis Ttl2; 9 – Bacillus sp. Stl7; 10 – B. thuringiensis B-6066.

Ten strains and isolates of the Bacillus spp. were tested for the presence of lipopeptide synthetases genes (Fig. 1).

As in B. subtilis 26D, in the strains of B. subtilis Tas2, Tas8.2 and Tas2.1, gene encoding surfactin synthetase srf was found (Fig. 1). As in the B. subtilis 11VM strain, in the B. subtilis Ttl2 strain and the Bacillus sp. Stl7 isolate, genes encoding iturin synthetase ituA and ituB and fengycin synthetase fenD were found, and in the strains of B. velezensis, TV2 and Ttl1, only genes encoding inturin synthetase were detected. The genes encoding surfactin and fengycin synthetase were identified in the B. thuringiensis B-6066 strain (Fig. 1).

Direct aphicidal effect of endophytic strains and isolates of bacteria Bacillus spp. and LRF on the S. graminum

Analysis of the aphicidal activity of ten strains and isolates of the genus Bacillus showed that all bacteria had high insecticidal activity against Greenbug aphid (Table 4).

**Table 4.**
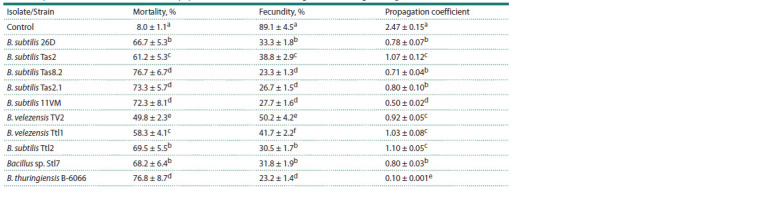
Aphicidal (insecticidal) effect of endophytic strains and isolates of the genus Bacillus against S. graminum Note. The same Latin letters in one column indicate that the values aren’t statistically different according to Duncan’s test (n = 30, p ≤ 0.05).

Aphid mortality increased from 8 to 50–77 % during feeding on bacterial suspension (Table 4). Accordingly, the fecundity of aphids decreased. In addition, bacteria reduced the propagation coefficient of aphids by 2–5 times (Table 4). The greatest aphicidal activity was shown by the B. subtilis 26D, B. subtilis 11VM, B. subtilis Ttl2, B. subtilis Tas2.1, B. subtilis Tas8.2 and B. thuringiensis B-6066 strains and the Bacillus sp. Stl7 isolate (Table 4).

**Fig. 2.**
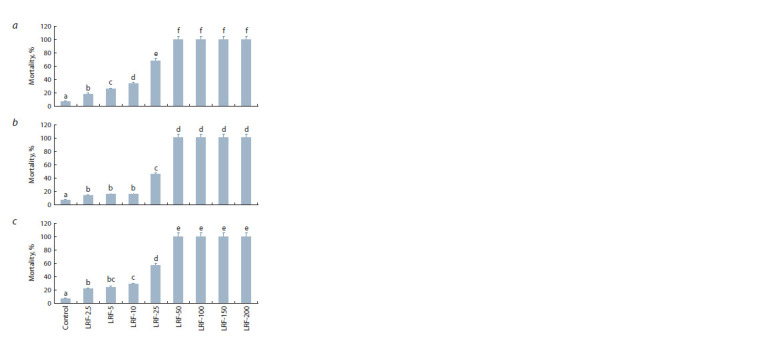
Aphicidal activity of LRFs of the strains B. subtilis Tas8.2 (а), B. subtilis Tas2.1 (b) and B. thuringiensis B-6066 (c) against Greenbug aphid S. graminum. Concentrations used for LRFs were 2.5, 5, 10, 25, 50, 100, 150 and 200 μg/ ml. Figures present means ± SE (n = 15). Columns of each histogram marked with the same Latin letters indicate that the values aren’t statistically different according to Duncan’s test ( p ≤ 0.05).

All studied strains synthesized lipopeptides (Fig. 1). To confirm the hypothesis about the role of lipopeptides in the aphicidal activity of Bacillus spp. LRFs were isolated from five strains. First of all, the aphicidal activity of LRFs was tested. The aphicidal activity of LRFs of the strains B. subtilis 26D (LRFBs26D) and B. subtilis 11VM (LRFBs11VM) was studied previously (Rumyantsev et al., 2023). And it was shown that the concentration of 25 μg/ml of LRFBs26D or LRFBs11VM caused the death of 50 % of aphids, and 100 % death of aphids was caused by 150 μg/ml already on the 5th day of feeding (Rumyantsev et al., 2023). LRFs of the strains B. subtilis Tas8.2, B. subtilis Tas2.1 and B. thuringiensis B-6066 (LRFBsTas8.2, LRFBsTas2.1 and LRFBt B-6066) as well as the strains themselves had a negative effect on the viability of S. graminum at direct influence (Fig. 2). The concentration of 25 μg/ml of LRFBsTas8.2 and LRFBt B-6066 caused death in more than 50 % of aphids, but not LRFBsTas2.1. However, 100 % of aphids died on the 5th day of feeding with solutions of LRFBsTas8.2, LRFBsTas2.1 and LRFBt B-6066 at a concentration of 50 μg/ml (Fig. 2).

The plant-mediated effect of endophytes of Bacillus spp. and LRF on various types of resistance (antibiosis, endurance) of wheat plants against S. graminum

In further study of the indirect effect of bacteria on the pest, seven strains and isolates were taken that showed the highest aphicidal activity, which are presented in Table 5. All seven bacterial strains and isolates had an indirect effect on aphid mortality and propagation coefficient. Aphid mortality increased from 10.9 to 36.3 % during aphid feeding on bacteria-treated plants (Table 5). Some bacteria reduced the propagation coefficient of aphids by 1.5–2 times (Table 5). The B. subtilis 26D and B. thuringiensis B-6066 strains and the Bacillus sp. Stl7 isolate had the greatest effect on aphid mortality, and the propagation coefficient was most strongly influenced by the B. subtilis 26D, B. subtilis Ttl2, B. subtilis Tas2.1 and B. subtilis Tas8.2 strains (Table 5). Moderate susceptible cv. SY showed low tolerance (endurance) to S. graminum, which manifested itself in inhibition of the growth of the first and second leaves in seedlings by 20 and 30 %, respectively (Table 5). Treatment of plants with bacterial strains and isolates increased plant resistance to Greenbug aphid by accelerating leaf growth by 10–20 % compared to the control and by 30–50 % compared to plants infested with aphids (Table 5).

**Table 5.**
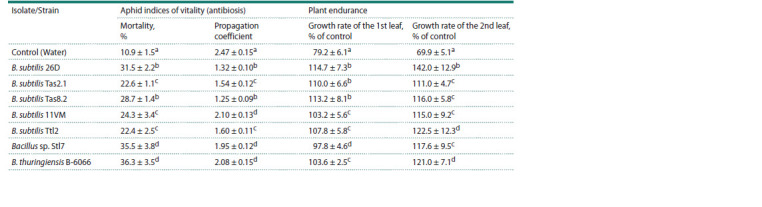
The effect of endophytes of Bacillus spp. on the vitality of aphids and endurance of S. graminum-infested wheat plants Note. Growth rate of the 1st or 2nd leaf of control, non-treated with bacterial strains and non-infested with aphids is 100 %. The same Latin letters in one column indicate that the values aren’t statistically different according to Duncan’s test (n = 30, p ≤ 0.05).

Since the effect of bacteria on plants and pests depends on the synthesis of various metabolites, we tested the indirect effect of LRF from five bacterial strains presented in Table 6 on the aphid indices of vitality and endurance of wheat plants

**Table 6.**
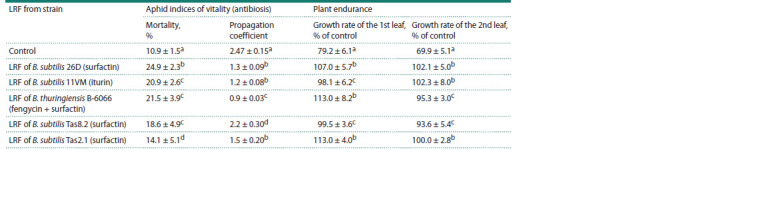
Effect of lipopeptide-rich fractions (LRFs) of three Bacillus spp. strains on the vitality of aphids and endurance of S. graminum-infested wheat plants Note. Growth rate of the 1st or 2nd leaf of control, non-treated with bacterial suspensions and non-populated with aphids is 100 %. The same Latin letters in one column indicate that the values aren’t statistically different according to Duncan’s test (n = 30, p ≤ 0.05).

Major lipopeptides in the LRFBs26D and LRFBs11VM were surfactin and iturin, respectively, which was confirmed by HPLC (Rumyantsev et al., 2023). LRFBt B-6066 presumably contained a mixture of fengycin and surfactin and LRFBsTas8.2, LRFBsTas2.1 contained surfactin (Fig. 1). Growth-promoting concentrations of LRFBs26D, LRFBs 11VM, LRFBt B-6066, LRFBsTas8.2, and LRFBsTas2.1 increased plant tolerance to the pest and increased aphid mortality, but to a lesser extent than bacterial strains (Table 6).

However, the propagation coefficient of aphids decreased much more during feeding on LRF-treated plants than on plants treated with the B. subtilis 11VM and B. thuringiensis B-6066 strains. LRFBt B-6066 had the greatest influence on the propagation coefficient of aphids, which indicates the role of fengycin in the indirect effect on aphid indices of vitality (Table 6). Thus, the results of this work show that lipopeptides, besides the direct insecticidal effect (Rumyantsev et al., 2023), manifest an indirect effect on the pest.

The plant-mediated effect of endophytes of Bacillus spp. and LRFs on changes in the redox status of S. graminum-infested wheat plants

The plant-mediated effect of endophytes of Bacillus spp. and their LRFs on plant endurance and indices of vitality of aphids may be connected with the start of induced systemic resistance (ISR) in plants (Rashid, Chung, 2017; Veselova et al., 2019). During the development of ISR, bacteria can affect the accumulation of ROS, both locally and systemically (Rashid, Chung, 2017).

**Fig. 3.**
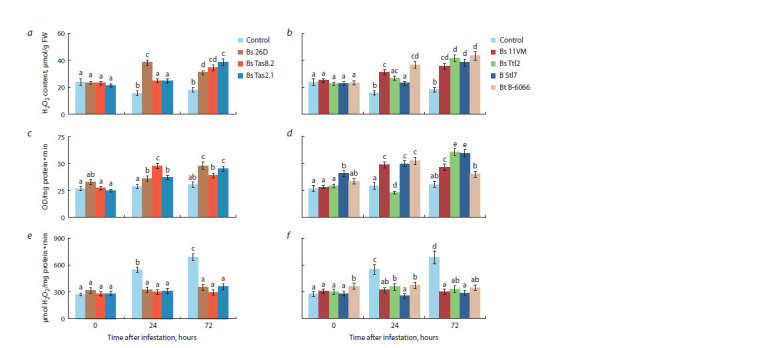
The effect of endophytes of Bacillus spp. on the content of hydrogen peroxide (H2O2) (a, b), activity of peroxidase (c, d ), and activity of catalase (e, f ) of S. graminum-infested wheat plants. Designations in the figure: 0 h – plants uninfested by aphids; Control – unbacterized plants; Bs 26D, Bs Tas8.2, Bs Tas2.1, Bs 11VM, Bs Ttl2, B Stl7 and Bt B-6066 – plants treated by the appropriate strain or isolate. Figures present means ± SE (n = 9). Columns of each histogram marked with the same Latin letters indicate that the values aren’t statistically different according to Duncan’s test ( p ≤ 0.05).

The infestation of non-bacterial control plants by aphids led to a decrease in the content of hydrogen peroxide (Fig. 3a, b), the absence of an increase in peroxidase activity (Fig. 3c, d ) and an increase in catalase activity (Fig. 3e, f ) 24 and 72 hours post aphid infestation and was accompanied by low aphid mortality and low plant endurance (Table 5). In wheat plants treated with strains and isolates of Bacillus spp. and infested with S. graminum, a sharp accumulation of H2O2, an increase in POD activity, no change in CAT activity compared to the control ones were found (Fig. 3).

The accumulation of H2O2 that was observed in bacterized plants of colonized aphids was associated with high pest mortality (Table 5, Fig. 3a, b). Treatment with strains B. subtilis 26D, B. subtilis 11VM, and B. thuringiensis B-6066 had the greatest effect on H2O2 accumulation 24 hours after aphid infestation. All strains and isolates equally increased the content of H2O2 after 72 hours post aphid infestation (Fig. 3a, b).

**Fig. 4.**
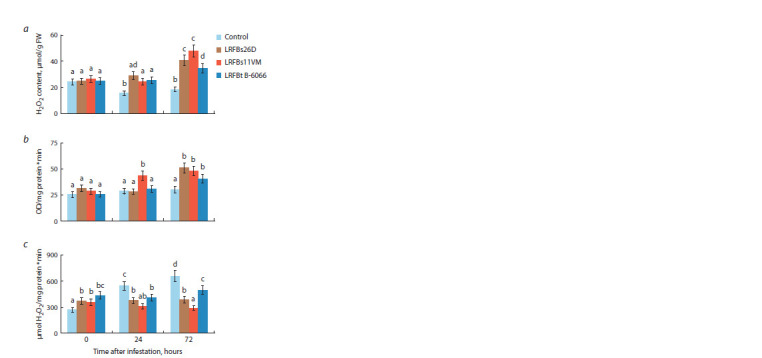
Effect of lipopeptide-rich fractions (LRFs) of the B. subtilis 26D (LRFBs26D), B. subtilis 11VM (LRFBs11VM) and B. thuringiensis B-6066 (LRFBt B-6066) strains on the content of hydrogen peroxide (H2O2) (a), activity of peroxidase (b), and activity of catalase (c) of S. graminum-infested wheat plants. Designations in the figure: 0 h – plants uninfested by aphids; Control – unbacterized plants; LRFBs26D, LRFBs11VM and LRFBt B-6066 – plants treated by the appropriate LRFs 24 h before aphid infestation. Figures present means ± SE (n = 9). Columns of each histogram marked with the same Latin letters indicate that the values aren’t statistically different according to Duncan’s test (p ≤ 0.05).

Treatment with strains B. thuringiensis B-6066, B. subtilis 11VM, B. subtilis Tas8.2 and Bacillus sp. Stl7 isolate increased POD activity earlier than treatment with strains of B. subtilis 26D, B. subtilis Ttl2 and B. subtilis Tas2.1 (Fig. 3c, d ). The first bacteria mentioned acted 24 hours after plant infestation by aphids, and the second bacteria mentioned activated POD 72 hours after plant infestation by aphids (Fig. 3c, d ). LRFs affected components of the pro-/antioxidant system of plants in the same way as bacterial strains (Fig. 4). However, LRFBs26D, LRFBt B-6066, and LRFBs11VM significantly induced the accumulation of H2O2 only 72 hours after plant colonization with the pest (Fig. 4a), unlike bacteria that induced H2O2 accumulation after 24 hours of feeding (Fig. 3).

LRFBs11VM and LRFBs26D increased POD activity in plants infested with aphids, as well as in plants treated with bacterial strains B. subtilis 11VM and B. subtilis 26D (Fig. 4b,Fig. 3). LRFBt B-6066 increased POD activity later than treatment with the bacterial strain, only 72 hours after aphid infestation (Fig. 4b). Treatment of wheat plants with LRFs did not lead to an increase in CAT activity during aphid feeding (Fig. 4c). Such results may indicate the possible role of lipopeptides in the induction of systemic resistance against Greenbug aphid in wheat. 

The plant-mediated effect of endophytes of Bacillus spp. and LRFs on changes in the expression of defense-related genes of PR proteins of wheat plants populated by S. graminum

Another indicator of the formation of systemic resistance in plants is considered to be an increase in the expression of defense-related genes of pathogenesis-related (PR) proteins, which is regulated by intermediate products of cell signaling systems (for example, H2O2) and phytohormones (Pieterse et al., 2014). The expression of defense-related Pr genes, salicylate (SA)-regulated and ethylene/jasmonate (JA)-regulated markers have been studied to test the bacteria-mediated activation of systemic resistance in S. graminum-infested plants. Proteins PR1, PR2 (glucanases) are markers of the SA signaling pathway. PR3 proteins (chitinases) are considered ethylene (ET)-regulated markers, and PR6 proteins (proteinase inhibitors) are considered JA-regulated markers. Proteins of PR9 (peroxidases) are both SA-responsive and JA-responsive pathogenesis- related proteins (Pieterse et al., 2014). In this work, in the moderately susceptible cv. SY, a slight increase of transcripts level of the Pr3 and Pr6 genes, markers of the ET- and JA-signaling pathways, respectively, and an increase of the expression levels of the Pr9 gene 72 hours after aphid colonization were found (Table 7).

**Table 7.**
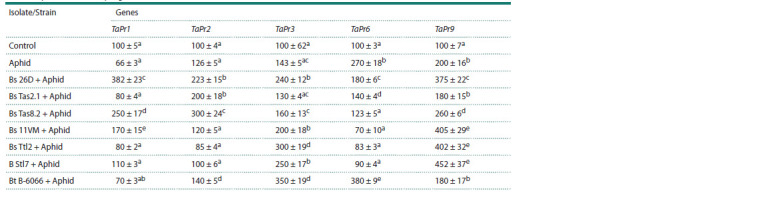
The effect of endophytes of the Bacillus spp. on changes in the expression of Pr genes of wheat plants infested by S. graminum Note. The same Latin letters in one column indicate that the values aren’t statistically different according to Duncan’s test (n = 9, p ≤ 0.05).

The effect of bacterial treatment on the expression of Pr genes had a different pattern. All seven bacterial strains and isolates increased the transcripts level of the Pr9 gene in aphid-infested plants compared to the control (Table 7). Six strains and isolates, excluding the B. subtilis Tas2.1 strain, increased the expression levels of the Pr3 gene, an ET-regulated marker, in S. graminum-infested plants. However, only two strains, B. thuringiensis B-6066 and B. subtilis Ttl2, influenced the expression levels of the Pr3 gene more strongly than others (Table 7).

Only four strains of B. subtilis 26D, 11VM, Tas8.2 and Tas2.1 significantly increased the expression levels of SA-regulated markers genes Pr1 and Pr2 in S. graminum-infested plants compared to the control. Only one strain of B. thuringiensis B-6066 increased the expression levels of the Pr6 gene, a marker of the JA-signaling pathway, in S. graminum-infested plants (Table 7).

**Fig. 5.**
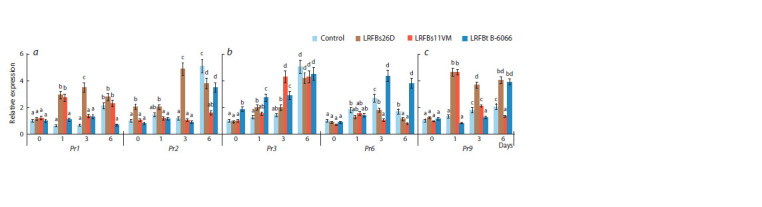
Effect of lipopeptide-rich fractions (LRFs) of the B. subtilis 26D (LRFBs26D), B. subtilis 11VM (LRFBs11VM) and B. thuringiensis B-6066 (LRFBt B-6066) strains on the relative expression of the Pr1 and Pr2 genes (a), Pr3 and Pr6 genes (b) and Pr9 gene (c) in S. graminum-infested wheat plants. Designations in the figure: 0 h – plants uninfested by aphids; Control – unbacterized plants. Figures present means ± SE (n = 9). Columns of each histogram marked with the same Latin letters indicate that the values aren’t statistically different according to Duncan’s test (p ≤ 0.05).

LRFs affected the expression of defense-related Pr genes of plants in the same way as bacterial strains, however, the degree of influence of LP was much higher (Fig. 5). Treatment with LRFBs26D, in which the major lipopeptide was surfactin, affected the accumulation of mRNA levels of the Pr1 and Pr2 genes in S. graminum-infested plants more than treatment with the B. subtilis 26D strain (Fig. 5a). Treatment with LRFBs11VM, in which the major lipopeptide was iturin and which also contained fengycin, increased the expression levels of the Pr1 and Pr3 genes in S. graminum-infested plants twice as much as treatment with the B. subtilis 11VM strain (Fig. 5a, b). The effect of LRFBt B-6066 on the expression of Pr genes resembled the effect of the B. thuringiensis B-6066 strain (Fig. 5, Table 7).

However, it is worth noting that LRFBt B-6066 contained two lipopeptides – surfactin and fengycin. Treatment with LRFBt B-6066 increased the transcripts level of the Pr3 gene as LRFBs11VM, increased the mRNA content of the Pr9 gene as LRFBs26D and, in addition, only LRFBt B-6066 affected the expression of the Pr6 gene in S. graminum-infested plants (Fig. 5). Importantly, the expression of some Pr genes induced by LRFs was activated later than during the treatment with the corresponding bacterial strains, 6 days after plant colonization by aphids (Fig. 5, Table 7). Thus, the results of this work show that lipopeptides have elicitor activity and induce the expression of defense-related Pr genes in aphid-infested plants.

## Discussion

In this research, ten endophyte isolates of the genus Bacillus from the collection of the Laboratory of Biochemistry of Plant Immunity of the Institute of Biochemistry and Genetics UFRC RAS were studied. Although the bacteria have been isolated from the inner tissues of various plants, many of them have been tested for their ability to colonize the inner tissues of other host plants (Veselova et al., 2019, 2022; Sorokan et al., 2020; Rumyantsev et al., 2023). All studied strains and isolates were found to have lipopeptide synthetase genes (Fig. 1) and all strains and isolates showed aphicidal activity (Table 4), which was due to the synthesis of lipopeptides as the results showed (Fig. 2).

In this work, using LRF isolated from Bacillus spp. strains, it was proven that the aphidicidal activity of bacterial strains against Greenbug aphid was due to lipopeptides – surfactin, iturin and fengycin (Fig. 2). This coincides with the results of other authors. Nowadays, the insecticidal activity of surfactin, plipastatin (fengycin family), bacillopeptin and iturin against some species of phloem-feeding insects has been shown (Rashid et al., 2018; Rodríguez et al., 2018; Denoirjean et al., 2022). Our studies have recently shown that surfactin and iturin exhibit aphicidal activity against Greenbug aphid (Rumyantsev et al., 2023). In addition, the results of our recent work showed that commercial surfactin (Sigma, USA) exhibited the same aphicidal activity as LRF from the B. subtilis 26D strain (Maksimov et al., 2020). In this work, the aphicidal effect of fengycin was demonstrated for the first time (Fig. 2).

This work also shows that bacterial strains, isolates, and LRFs of three Bacillus spp. strains had an indirect effect on the indices of vitality of aphids and endurance of S. graminumpopulated wheat plants (Tables 5, 6). The weaker effect of bacteria on the mortality of aphids under the indirect effect compared to the direct effect was possibly due to the dif- ferent degree of plant tissue colonization of the strains and isolates, which we showed in another work using the B. subtilis 11VM strain as an example (Rumyantsev et al., 2023). Thus, when testing for endophyticity, the B. subtilis 26D strain showed the greatest ability to reproduce in the plant tissues, the other strains and isolates studied in this work reproduced in the tissues of plants by an order of magnitude less, and the Bacillus sp. Stl7 isolate reduced the number of cells by two orders of quantity compared to the B. subtilis 26D strain (Veselova et al., 2019, 2022; Sorokan et al., 2020; Rumyantsev et al., 2023).

LRFs increased plant tolerance, but to a weaker extent than bacterial strains and isolates (Tables 3, 4). The influence of bacteria on plant growth may be associated with the synthesis of hormone-like compounds by bacteria and the effect on the availability of nutrients for plants (Eid et al., 2021). Also, the effect on plant growth may be indirect through the synthesis of metabolites with biocidal activity, which reduce the infection load on plants, and may trigger systemic resistance in plants (Eid et al., 2021). Presumably, the effect of LRFs on plant growth was indirect and was related to the stimulation of systemic resistance in plants.

Verification of the indirect action of bacterial strains and isolates and LRFs showed that both bacteria and LRFs are able to change the redox status of plants inhabited by aphids (Fig. 3, 4) and cause an oxidative burst, which subsequently induces the expression of defense-related Pr genes (Rashid et al., 2018; Tunsagool et al., 2019; Oukala et al., 2021). Thus, the generation of ROS during attack by phloem-feeding insects is discussed as a resistance response against pests (Koch et al., 2016; Veselova et al., 2019). The jump in the ROS generation, including H2O2, can lead both to direct damage to aphids and their death, and to the circumstantial effect of H2O2 through signaling regulation of resistance and gene expression (Rashid, Chung, 2017; Rashid et al., 2018). In addition, bacterial strains and isolates, and LRFs affected the activity of redox enzymes – POD and CAT in aphid-infested plants (Fig. 3, 4). Low catalase activity was found in aphid resistant crop phenotypes (Zhu-Salzman et al., 2004). An increase in POD activity under the influence of bacteria led to an improvement in the strategy of plant resistance against insects (Rashid et al., 2018; Veselova et al., 2019; Ling et al., 2022). To date, the role of lipopeptides in the regulation of ROS generation and the work of redox enzymes has been studied only during infection of plants with pathogenic fungi (Farzand et al., 2019; Tunsagool et al., 2019). These works showed the positive effect of fengycin, surfactin and iturin on the activity of peroxidases in plants during the attack of fungal pathogens (Farzand et al., 2019; Tunsagool et al., 2019). This work demonstrates for the first time the ability of strains and isolates B. subtilis Ttl2, Bacillus sp. Stl7 and B. thuringiensis B-6066, which synthesize fengycin, regulate components of the pro-/antioxidant system of aphid-infested plants.

Bacterial strains and isolates and LRFs induced the expression of defense-related Pr genes, markers of hormonal signaling pathways such as JA, SA and ethylene (Table 7, Fig. 5). All three hormonal signaling pathways are known to play a role in plant defense against phloem-feeding insects and other pests (Morkunas et al., 2011; Pangesti et al., 2016). B. subtilis induced resistance against the whitefly Bemisia tabaci on tomato plants by activating SA- and JA-responsive genes. Rhizobacteria Pseudomonas simiae WCS417r induced Arabidopsis defense reaction against Mamestra brassicae by activating the synthesis of camalexin and aliphatic glucosinolates, which is regulated by the ORA59-branch of the JA/ ethylene signaling pathway (Pangesti et al., 2016). A series of studies have shown that the ethylene signaling pathway is required for the polymerization of phloem proteins, which block phloem pores and therefore prevent aphids feeding (Fu et al., 2014; Lu et al., 2023).

Unfortunately, there are very few works on the activation of resistance against insects by lipopeptides. Thus, it was shown that the bacillopeptin of the B. velezensis YC7010 strain, which induces the deposition of lignin and callose in plants, increased the resistance of rice against Nilaparvata lugens (brown planthopper) (Rashid et al., 2018). Nowadays, the role of lipopeptides in the activation of plant resistance against various pathogens through the induction of JA/ethylene-, ABA-, SA- and auxin-dependent response is well studied (Tunsagool et al., 2019; Jiang et al., 2021). Our results showed that lipopeptides surfactin, fengycin and iturin activated the expression of defense-related Pr genes of the SA-, JA- and ethylene-regulated markers in wheat against the S. graminum. Our results suggest a role of fengycin in inducing the expression of ethylene-dependent genes (Fig. 5), which is consistent with results obtained during studies of resistance to pathogen (Waewthongrak et al., 2014). This work demonstrates for the first time the elicitor role of fengycin in triggering the systemic resistance of wheat plants against S. graminum.

## Conclusion

In the ten studied strains and isolates of endophytes of the genus Bacillus, lipopeptide synthetase genes were found, and all bacteria had aphicidal activity. This study shows that lipopeptides play a role in the defense of plants from phloemfeeding insects through a direct and an indirect mechanism of action. We have discovered new promising strains and isolates of endophytes of the genus Bacillus, which can become the basis for future biocontrol agents against aphids. The search for new bacteria active against phloem-feeding insects can be conducted by the presence of lipopeptide synthetase genes in the bacterial genome.

## Conflict of interest

The authors declare no conflict of interest.
